# Focused-Ion-Beam Artifacts and Evidence Reliability in Advanced Microscopy of Energy Materials

**DOI:** 10.3390/molecules31122148

**Published:** 2026-06-18

**Authors:** Chen Chen, Liangjuan Gao, Jiaqi Jia, Zhao Ding

**Affiliations:** 1 Department of Mechanics, Jinzhong University, Jinzhong 030606, China; chenchentgzy@163.com; 2 College of Materials Science and Engineering, Sichuan University, Chengdu 610065, China; lgao87@scu.edu.cn; 3 College of Materials Science and Engineering, National Engineering Research Center for Magnesium Alloys, Chongqing University, Chongqing 400044, China; cq-jiaqi@cqu.edu.cn

**Keywords:** focused ion beam, FIB-SEM, energy materials, microscopy artifacts, cryo-FIB, solid-state electrolytes, reactive interfaces, FIB-SEM tomography, claim-specific validation

## Abstract

Focused-ion-beam scanning electron microscopy (FIB-SEM) provides site-specific access to buried interfaces, particle interiors, porous electrode architectures, and localized degradation regions in energy materials. This capability is particularly valuable for rechargeable batteries, solid-state ion conductors, alkali-metal electrodes, and reactive solid–liquid interfaces, where the structures governing transport and failure are rarely exposed at a free surface. However, the preparation and imaging steps that reveal these regions may also alter them. Ion milling, environmental transfer, vacuum exposure, scanning electron microscopy (SEM), cryogenic handling, transmission electron microscopy (TEM), scanning transmission electron microscopy (STEM), energy-dispersive X-ray spectroscopy (EDS), electron energy-loss spectroscopy (EELS), and atom probe tomography (APT) can each modify local morphology, chemistry, or phase state. These effects are especially important when the intended evidence involves light elements, metastable phases, nanoscale coatings, reactive interphases, volatile species, or ion-conducting materials. This perspective develops a claim-specific framework for evaluating such results. Preparation- and imaging-induced changes are related to the material feature being interpreted and to the minimum control needed to distinguish the two origins. For porous electrodes, the relevant outputs include pore volume, connectivity, tortuosity, crack geometry, phase fraction, and active surface area. For reactive interfaces and solid electrolytes, the critical questions concern alkali-metal redistribution, surface amorphization, light-element contrast, implanted-species chemistry, and beam-induced phase formation. The discussion further compares conventional Ga-FIB, cryogenic FIB, Xe plasma FIB, low-energy Ar+ polishing, broad-ion-beam preparation, ultramicrotomy, and repeated particle-oriented FIB workflows. Reliable interpretation requires the preparation route, transfer conditions, imaging dose, analytical acquisition, and claim-specific controls to be reported together with the final microscopy result.

## 1. Introduction

Energy materials increasingly require microscopy evidence that is local, chemically specific, and tied to device operation. In rechargeable batteries, solid-state ion conductors, electrocatalytic interfaces, fuel-cell components, and porous composite electrodes, the decisive structural features are rarely exposed on a clean free surface. They are often buried at solid–solid contacts, distributed through composite electrode networks, confined at solid–liquid interfaces, or located inside secondary particles where coating layers, cracks, grain boundaries, and reaction products coexist over nanometer-to-micrometer length scales. Performance and failure are therefore inferred from evidence that is difficult to access directly: a cathode surface reconstruction layer only a few nanometers thick, a chemically heterogeneous solid-electrolyte interphase, a lithium- or sodium-rich filament, a space-charge-modified solid-electrolyte boundary, or a local pore pathway that controls ion transport. For these systems, microscopy does not merely record morphology. It often supplies the structural and chemical basis on which transport, degradation, and interfacial reaction mechanisms are proposed.

Focused-ion-beam scanning electron microscopy (FIB-SEM) has become one of the most widely used routes for gaining this access in battery and energy-materials microscopy [1]. In a single platform, FIB-SEM combines site-specific milling, electron-beam imaging, local deposition, cross-sectioning, serial sectioning, and preparation of electron-transparent lamellae for subsequent transmission electron microscopy (TEM) and scanning transmission electron microscopy (STEM) analysis [2,3,4]. Recent battery studies have used FIB-SEM to examine nanoscale interphases, cracking and reconstruction within individual particles, three-dimensional electrode architectures, and degradation across multiple length scales. Cryogenic workflows have further expanded its use to alkali metals, solid-electrolyte interphases, solid-state electrolytes, and liquid-containing interfaces that would otherwise be altered during conventional preparation and transfer. FIB-SEM is therefore no longer only a specimen-preparation method. It now provides a practical link between site-specific specimen preparation and structural or chemical analysis across multiple length scales.

The same capability defines the central interpretational problem. FIB-based characterization creates access by modifying the specimen, a limitation long recognized in TEM lamella preparation and FIB-induced damage studies [5,6]. During preparation, the region of interest may be exposed, sputtered, implanted, redeposited upon, heated, charged, thinned, polished, or protected by deposited layers [7,8]. During subsequent imaging and spectroscopy, the same region may experience electron-beam-induced charging, radiolysis, knock-on damage, hydrocarbon contamination, phase transformation, or time-dependent changes in elemental intensity and spatial distribution. For many stable ceramics or engineering alloys, these effects may remain secondary to the structural information sought. For energy materials, they can overlap directly with the signals used to interpret function. Lithium and sodium redistribution, interfacial oxidation, surface amorphization, local phase conversion, or nanoscale enrichment of an implanted species may be mistaken for electrochemical degradation, transport-induced segregation, or an intrinsic interphase.

**Figure 1:**
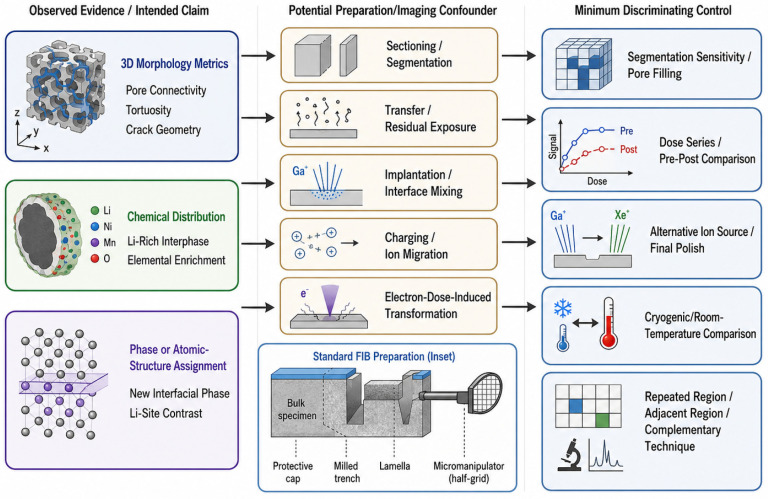
Claim-specific assessment of microscopy evidence in energy materials. Representative structural and chemical claims are paired with preparation- or imaging-induced changes that may produce similar observations and with controls that can distinguish intrinsic material features from workflow-induced alterations. The inset depicts a conventional site-specific FIB lift-out sequence in which a protected lamella is trench-milled, undercut, attached to a micromanipulator needle, transferred to a half-grid, locally welded using Pt or C deposition, and subsequently thinned.

Reliable interpretation requires the origin of an observed feature to be evaluated across the complete preparation and imaging procedure. Damage observed after TEM or STEM analysis should not be assigned automatically to FIB preparation. Air exposure and solvent removal can modify reactive or liquid-containing interfaces before the specimen reaches the microscope. Ion-beam milling can introduce amorphous layers, implanted ions, redeposited material, and local phase mixing [5,6,7,8]. SEM navigation can induce charging and alkali-metal migration in insulating solid electrolytes [9]. TEM, STEM, electron energy-loss spectroscopy (EELS), and energy-dispersive X-ray spectroscopy (EDS) can introduce additional dose-dependent transformations during imaging or acquisition. The most serious artifact is not always the most visually obvious one; it is often the one that appears chemically meaningful. Figure 1 relates representative structural and chemical claims to preparation- or imaging-induced changes that may produce similar observations and to the controls needed to distinguish them.

The purpose of this perspective is not to restate the familiar principle that specimen preparation can affect microscopy results. It addresses a narrower problem that is especially important for energy materials: preparation or imaging may generate the same type of evidence that would otherwise be assigned to electrochemical transport, degradation, or interfacial reaction. An altered pore network may be interpreted as an intrinsic transport architecture; a beam-induced alkali-metal protrusion may resemble a filament formed during cycling; implanted species or interface mixing may appear as a new reaction phase; and a preparation-induced amorphous layer may obscure or redistribute the weak contrast used to locate light elements.

The discussion is therefore organized around the scientific claim rather than around an exhaustive catalogue of instrument-related artifacts. For each class of result, three questions are considered: which measured feature supports the proposed materials interpretation; which handling, milling, imaging, or analytical process could produce a similar feature; and which minimum control is needed to distinguish an intrinsic material response from a preparation- or dose-induced change. This claim-specific approach is applied to quantitative descriptors of electrode morphology, including pore volume, connectivity, tortuosity, and crack geometry; to reactive interphases and alkali-metal redistribution; to light-element and atomic-structure imaging; and to the assignment of new interfacial phases or elemental enrichment. The resulting validation and reporting requirements are summarized in Table 1.

## 2. Sources of Specimen Alteration During Handling, Preparation, Imaging, and Analysis

For energy materials, specimen alteration may begin before ion milling. A cathode particle, solid-electrolyte interface, lithium-metal deposit, sulfide or halide electrolyte, or porous composite electrode may already have undergone air or moisture exposure, solvent removal, vacuum transfer, protective-layer deposition, SEM observation, or cryogenic handling before the final image or spectrum is acquired [10,11]. The relevant question is therefore not simply whether FIB damaged the specimen, but which stage of preparation or observation created or modified the feature being interpreted.

Changes may be introduced during four closely connected stages: environmental handling and transfer, FIB preparation, SEM imaging, and TEM/STEM imaging or spectroscopic analysis. These stages cannot always be considered independently. FIB milling exposes fresh surfaces that may react with residual gases; SEM navigation may charge an insulating region before ion milling is complete [9]; and TEM/STEM analysis acts on a lamella that has already been thinned, supported, irradiated, and possibly chemically modified. The final observation may therefore reflect the cumulative effect of several small changes rather than a single identifiable damage event.

Environmental and transfer artifacts are especially relevant for reactive energy materials. Solid–liquid interfaces, lithium-metal surfaces, SEI layers, and some sulfide or halide electrolytes may change during air exposure, solvent removal, drying, vacuum transfer, or warming [10,11]. Cryogenic FIB/SEM can suppress diffusion, evaporation, and chemical reaction during transfer, sectioning, and mapping, but it does not erase the specimen’s preparation history. Freezing quality, transfer integrity, residual chamber gases, local warming, ice contamination, and electron dose can still modify the final state. Cryogenic data should therefore be interpreted as measurements obtained under a defined low-temperature preparation protocol rather than as an automatically pristine representation of the original material.

FIB preparation remains a major source of alteration because ion-beam milling directly modifies the material subsequently examined by TEM, STEM, diffraction, or spectroscopy. Conventional Ga-FIB can produce amorphous surface layers, Ga implantation, collision cascades, sputtering, redeposition, roughness, and local phase mixing. In dense oxide or metallic systems, these changes may be tolerable for some morphological questions but damaging for others. In solid electrolytes, for example, a FIB-induced amorphous layer can obscure weak light-element signals and reduce confidence in annular bright-field scanning transmission electron microscopy (ABF-STEM) or integrated differential phase-contrast STEM (iDPC-STEM) interpretation of lithium positions. In metal-containing interfaces, implanted species or local mixing may generate features that look crystallographically and chemically meaningful. FIB damage should therefore be evaluated according to the signal being claimed, not only according to whether the lamella appears smooth or electron-transparent.

Protective deposition, lamella attachment, and final thinning introduce additional variables that are not captured by ion-beam damage alone. Electron- or ion-beam-induced C or Pt layers can protect a surface from direct sputtering and reduce curtaining, but they may also obscure nanometer-scale surface features, contribute C or Pt signals to later spectroscopy, or mask the true termination of a coating or interphase. During lift-out, the lamella is normally attached to a Cu or Mo half-grid or post by local C or Pt deposition. The attachment position and weld geometry impose mechanical constraints that become more important as the lamella is thinned.

Final thinning reduces specimen stiffness and may lead to bending, warping, stress relaxation, or opening and closure of pre-existing cracks. In strained crystalline or multilayer specimens, these changes may also modify the local strain field or the apparent dislocation arrangement. Such effects are particularly relevant when the conclusion concerns crack width, coating continuity, interfacial separation, lattice strain, or dislocation density. The cap material and thickness, grid type, attachment geometry, weld position, final lamella thickness and thickness gradient, and low-voltage cleaning conditions should therefore be reported when these quantities are central to the interpretation.

SEM imaging is not a passive step in this workflow. It is commonly used for survey imaging, region selection, trench monitoring, lift-out alignment, and final lamella inspection, but in insulating or ion-conducting energy materials the electron beam can itself modify the specimen. In solid-state electrolytes, Li or Na whisker growth can be induced by both electron and Ga^+^ ion beams at relatively low dose, together with changes in chemical composition. The effect has been linked mainly to surface charging and can be suppressed by Au coating or cryogenic preparation. Such beam-induced morphology can resemble electrochemical alkali accumulation or filament formation. If SEM navigation has already changed the local ion distribution, a feature subsequently observed by TEM cannot be assigned solely to FIB preparation or electrochemical cycling.

TEM and STEM add another level of ambiguity because they impose a different dose regime. Battery materials and their interfaces are often rich in light elements, chemically active, and sensitive to knock-on displacement, radiolysis, beam heating, electrostatic charging, and contamination. Advanced STEM and analytical mapping may require repeated scanning, long dwell times, or high probe current over regions that have already been thinned by FIB. The resulting transformation may be dose-dependent rather than preparation-dependent. Cryogenic electron microscopy can improve dose tolerance, but cooling does not eliminate beam-driven structural or chemical change. A phase observed after STEM imaging or EELS/EDS mapping cannot be assigned confidently to cycling or FIB preparation unless the electron-dose history has also been considered.

Analytical acquisition deserves separate attention because spectroscopy often supplies the evidence used to support interfacial chemistry. EDS and EELS are used to justify claims about elemental enrichment, oxygen loss, transition-metal migration, lithium depletion, SEI composition, or local decomposition. Yet these are also the signals most likely to evolve under prolonged acquisition. Increasing dwell time or frame integration may improve the signal-to-noise ratio while also increasing radiolysis, heating, contamination, beam-driven segregation, or time-dependent changes in elemental intensity and spatial distribution during acquisition. For low-Z or volatile components, the measured map may represent a compromise between intrinsic composition and dose-induced evolution during acquisition. A single high-quality elemental map is therefore not sufficient unless the acquisition history and possible beam response are constrained.

Carbon contamination is another practical source of alteration during SEM and TEM observation. Residual hydrocarbons in the microscope column or on the specimen can polymerize under electron irradiation and form a carbonaceous deposit over the scanned region. The deposited layer may increase the apparent specimen thickness, obscure weak lattice or light-element contrast, introduce an additional carbon signal, and progressively degrade EDS or EELS measurements. Because the deposit develops with exposure time and scanned area, its distribution may also be mistaken for a native carbon-rich surface layer or interphase.

Plasma cleaning of the holder, grid, or lamella can reduce hydrocarbon contamination, but it should not be treated as chemically neutral for all energy materials. Gas composition, power, cleaning time, and the interval between cleaning and analysis determine the resulting surface condition. Oxygen-containing plasma may be unsuitable for oxidation-sensitive metals, reactive SEI layers, and some sulfide or halide electrolytes unless the effect has been validated on a sacrificial or reference region. Cleaning conditions and any observable changes before and after treatment should therefore be reported when surface chemistry or carbon-containing interphases are interpreted.

Controls should be selected according to the feature being interpreted. A generally lower-dose FIB recipe does not by itself establish that a result is intrinsic. A claim concerning pore connectivity or tortuosity should be tested against slice-thickness variation, curtaining, redeposition, contrast generation, and segmentation sensitivity. A Li- or Na-rich interphase should be examined by dose-series or pre-/post-imaging comparisons and, where appropriate, by comparing cryogenic and room-temperature preparation. A newly identified interfacial phase should be checked against implanted-species chemistry, local mixing, final-polishing conditions, and an adjacent or alternatively prepared region. Similarly, weak atomic-column contrast should be evaluated against lamella thickness, surface amorphization, contamination, and electron dose. The appropriate control is therefore determined by the observation used to support the materials interpretation. A result becomes persuasive when the preparation or imaging process most capable of reproducing the same feature has been directly constrained.

## 3. Physical Artifacts in Sectioning, Tomography, and 3D Morphology Metrics

Physical artifacts are the most visible consequence of FIB processing, but their significance extends beyond image quality when sectioned or reconstructed data are used to report three-dimensional morphology metrics. Curtaining, redeposition, edge rounding, swelling, surface roughness, and nonuniform material removal can alter pore volume, phase fraction, connectivity, tortuosity, crack geometry, coating continuity, and active surface area [12,13]. These parameters are not equally sensitive to the same source of error. Pore volume and phase fraction depend strongly on contrast generation and segmentation; connectivity and tortuosity are additionally affected by slice spacing, registration, redeposition, and voxel anisotropy; crack width and coating continuity are particularly sensitive to edge rounding, overmilling, shadowing, and protective-cap geometry. The required control should therefore be selected according to the morphology parameter being reported. This issue is especially acute in porous battery electrodes, where electrochemical behavior depends on the three-dimensional arrangement of active particles, carbon-binder domains, electrolyte-filled pores, cracks, and interparticle contacts [14,15,16]. Pore connectivity and tortuosity influence ion transport; carbon-binder distribution affects electronic conduction; crack networks alter local reaction area and mechanical stability. FIB-SEM tomography is attractive because it can resolve electrode architectures at length scales that are difficult to access by conventional X-ray tomography. However, the same serial sectioning process that creates the 3D dataset also defines the accuracy of the reconstructed morphology. Slice-thickness variation, curtaining, drift, local charging, surface roughness, and redeposition may introduce structured errors that are carried through segmentation and modeling. For claims based on pore connectivity or tortuosity, robustness should therefore be tested against slice-thickness variation, registration quality, segmentation threshold, and, where possible, repeated or independently selected volumes.

**Figure 2:**
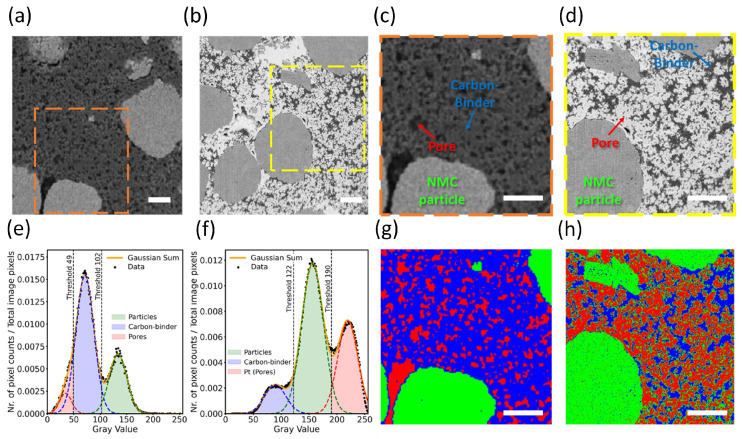
FIB-SEM tomography of porous battery electrodes without and with in situ Pt pore filling. (**a**) BSE-SEM image of the electrode cross-section without pore filling. (**b**) BSE-SEM image after in situ Pt pore filling. (**c**,**d**) Enlarged regions from (**a**) and (**b**), respectively, showing the contrast among active particles, carbon-binder domains, and pores. (**e**,**f**) Gray-value histograms and fitted phase distributions obtained from the images in (**a**) and (**b**), respectively. The green, blue, and red fitted components correspond to particles, carbon-binder domains, and pore-related regions, respectively; in (**f**), the red component represents Pt-filled pores. The orange curve denotes the Gaussian-sum fit, black dots denote the measured histogram data, and vertical dashed lines indicate the gray-value thresholds used for segmentation. (**g**,**h**) Segmentation maps derived from the enlarged images in (**c**,**d**) using the corresponding fitted distributions in (**e**,**f**). The partial overlap among fitted components in (**e**,**f**) reflects gray-value overlap between constituents and does not affect scientific understanding; rather, it illustrates the segmentation uncertainty that is reduced after Pt pore filling. Scale bars: 2 μm. Adapted with permission from Ref. [20].

The most vulnerable step is often not milling alone, but phase assignment. Active particles are usually easier to segment because of their stronger compositional contrast. Carbon-binder domains and pores are more difficult because their contrast can be weak, spatially variable, and sensitive to detector configuration, beam condition, topography, and charging [17,18,19]. In situ Pt pore filling has been used to enhance contrast in FIB-SEM tomography of porous battery electrodes [20]. Without pore filling, gray-value overlap between pores and carbon-binder domains makes segmentation sensitive to fitting assumptions. After Pt filling, the pore phase becomes more distinguishable, improving confidence in pore identification and phase-volume estimation (Figure 2). Contrast generation is therefore part of the measurement rather than a neutral preprocessing step. A pore-volume claim should be accompanied by threshold-sensitivity and pore-filling-completeness tests, whereas connectivity or tortuosity should also be checked for stability under alternative segmentation and registration conditions.

A tomography dataset also accumulates small sectioning errors over many slices. Curtaining can create artificial vertical intensity bands that resemble elongated pores or binder-rich domains. Redeposition can partially fill open pores and cause them to be segmented as solid material. Preferential sputtering can distort soft domains relative to dense particles [21,22]. Edge rounding can blur particle boundaries and reduce the apparent width of cracks. Slice-thickness variation can distort the z-axis dimension of pores and cracks, thereby changing connectivity. These effects may be modest in a single image, but they can be amplified when hundreds of slices are reconstructed into a 3D volume and then used for transport simulation or representative-volume analysis. Classic nanoscale FIB studies remain useful for identifying the physical mechanisms behind these errors. Silicon milling studies showed that Ga^+^ irradiation can produce swelling through amorphization at low dose, sputtering at higher dose, and geometry-dependent effects such as self-focusing, oblique-angle milling, aspect-ratio-dependent removal, and redeposition. These mechanisms explain why FIB does not remove material uniformly from complex topographies. In porous electrodes, cracked particles, rough coatings, and high-aspect-ratio pores, the same dependence on local angle and shadowing can change the apparent geometry of the measured feature.

Particle-level cross-sections raise a related problem. FIB is widely used to expose coatings, reconstructed surface layers, intragranular cracks, grain boundaries, and core–shell structures in secondary particles. These cross-sections are often interpreted as direct evidence of degradation or coating integrity. Yet a coating that appears discontinuous may have been locally thinned or removed by overmilling; a crack may appear wider because of edge rounding or shadowing; a thin surface layer may be hidden by redeposition or protective-cap geometry. High-throughput FIB workflows for microscale cathode particles improve statistical access and reduce preparation time, but they do not remove the need to report particle orientation, support geometry, lamella thickness, and repeated-particle consistency when coating continuity or internal composition is interpreted.

Representativeness is another source of physical uncertainty. FIB-SEM provides high spatial resolution over limited volumes, whereas battery electrodes are heterogeneous over length scales from nanometers to hundreds of micrometers. A small high-resolution volume may be ideal for resolving carbon-binder-pore morphology but insufficient for electrode-level heterogeneity. A larger volume improves representativeness but increases milling time, drift, curtaining risk, charging, and segmentation burden. This trade-off is not only technical. It determines the scale at which the conclusion is valid. A local pore-network reconstruction should not be used to support electrode-scale transport claims unless the volume is representative or explicitly linked to a larger-scale measurement. For this reason, sectioning and segmentation artifacts should be reported as sources of uncertainty in the specific 3D morphology metric being claimed. In FIB-SEM tomography, relevant descriptors include slice thickness, voxel anisotropy, curtaining amplitude, drift correction, redeposition, surface roughness, pore-filling completeness, and segmentation-threshold sensitivity. In particle cross-sections, they include final lamella thickness, milling angle, protective-cap geometry, edge rounding, damaged-layer thickness, and consistency across adjacent sections or repeated particles. These descriptors do not need to become an exhaustive checklist in every study, but they should match the parameter being claimed. A study reporting pore tortuosity should constrain segmentation and connectivity uncertainty; a study reporting coating coverage should constrain edge damage and cap-induced masking; a study reporting crack networks should constrain milling geometry and slice alignment.

Physical artifacts also influence chemical interpretation. Redeposited material can create apparent compositional overlap between adjacent domains. Roughness can broaden an interface in projection. Curtaining can distort the geometry of a region later analyzed by EDS or EELS. A pore wall damaged during milling may later be interpreted as a reaction layer. The separation between physical and chemical artifacts is therefore useful analytically, but not absolute experimentally. In energy electrodes, morphology and chemistry are often interpreted together, and geometric distortion can influence the chemical conclusion drawn from that geometry. Physical artifacts can therefore create false morphology–performance relationships: reconstructed electrode volumes may appear plausible while retaining biased connectivity, particle cross-sections may contain real cracks and coatings distorted by milling geometry, and segmented pore networks may support transport simulations while remaining sensitive to contrast and thresholding. A morphology-based conclusion becomes persuasive only when the sectioning, contrast, and segmentation choices most capable of altering the reported descriptor have been tested explicitly.

## 4. Chemical and Phase Artifacts in Reactive Energy Materials

Chemical and phase artifacts are more difficult to recognize than physical artifacts because they can appear as meaningful evidence. In Li-metal and SEI-containing systems, cryogenic electron microscopy has shown that nanoscale interphase structure and chemistry can be interpreted only when preparation and dose history are controlled together [23,24]. Reactive interfaces provide the clearest example. Lithium and sodium metals, solid-electrolyte interphases, solid–liquid interfaces, sulfide or halide electrolytes, and oxygen-deficient oxides can respond to air, residual gases, solvent removal, protective-layer deposition, ion beams, and electron beams. The resulting changes may be localized and chemically organized. The central difficulty is that preparation- or beam-induced change may reproduce the same chemistry or phase contrast expected from electrochemical operation. The appropriate control therefore depends on the assignment being made: reactive-interphase chemistry requires constraints on transfer and residual exposure; alkali-metal redistribution requires charging and dose controls; light-element or atomic-column interpretation requires control of surface damage, thickness, contamination, and dose; and a newly identified interfacial phase requires tests for implanted species, interface mixing, and beam-induced transformation.

**Figure 3:**
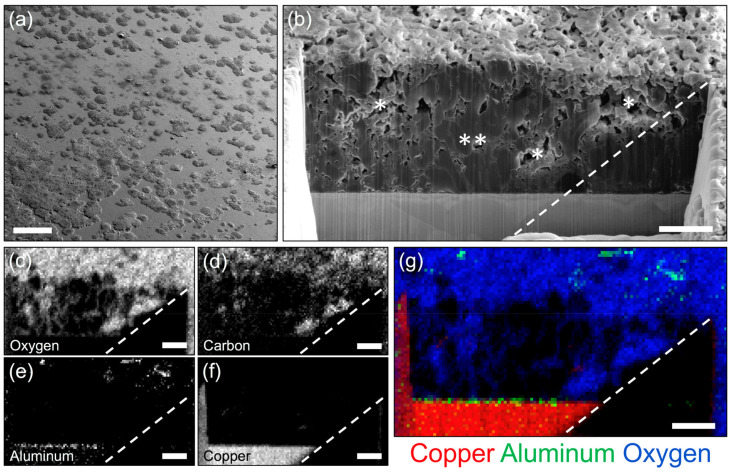
Cryo-FIB/SEM and cryo-EDX mapping of lithium deposits on an artificial SEI-coated copper current collector. (**a**) Cryo-SEM image of lithium deposited on a copper current collector coated with an approximately 15 nm alumina artificial SEI layer. (**b**) Cross-sectional cryo-FIB image showing dense and porous regions within the lithium deposits; ** marks dense lithium-deposition regions, whereas * marks porous lithium-deposition regions. (**c**–**g**) Cryogenic energy-dispersive X-ray spectroscopy (cryo-EDX) maps of the milled cross-section, resolving spatial variations in oxygen, carbon, aluminum, and copper signals. Dense regions show weak elemental contrast, whereas porous regions show enhanced carbon and oxygen signals. Dashed lines indicate detector-related shadowing in the EDX maps. Scale bars: (**a**) 300 μm; (**b**–**g**) 5 μm. Adapted with permission from Ref. [26].

Alkali-metal electrodes and SEI layers are especially vulnerable because their native chemistry is difficult to preserve outside the electrochemical environment [25]. Cryo-FIB/SEM offers a route to reduce these changes by freezing the electrochemical state and maintaining low temperature during transfer, milling, and analysis [26]. In lithium-deposition systems, cryogenic FIB milling and EDX mapping can distinguish dense lithium-rich regions from porous regions containing carbon- and oxygen-bearing species on an artificial-SEI-coated current collector (Figure 3). A Li-rich or oxygen- and carbon-containing interphase should therefore not be assigned from a single cryogenic map alone. At minimum, the transfer environment and exposure time should be documented, and the stability of the mapped feature should be checked by a low-dose sequence or pre-/post-acquisition comparison.

Cryogenic preparation does not erase the specimen’s handling or preparation history. Its purpose is to suppress selected diffusion, evaporation, and reaction pathways during transfer, milling, and analysis [27,28]. The final state may still depend on the freezing procedure, transfer time, residual chamber gases, local warming, ice contamination, and electron dose. In cryogenic plasma focused-ion-beam/scanning electron microscopy (PFIB/SEM)-prepared lithium lamellae, metallic lithium may remain structurally preserved during milling while Li_2_O diffraction features are still present in the final specimen [29]. Because the examined lamellae did not contact liquid oxygen or liquid nitrogen, the oxide was attributed mainly to the cryo-FIB/SEM chamber environment, although a transfer contribution could not be excluded. A structurally usable lamella may therefore still carry a chemically modified surface layer.

**Figure 4:**
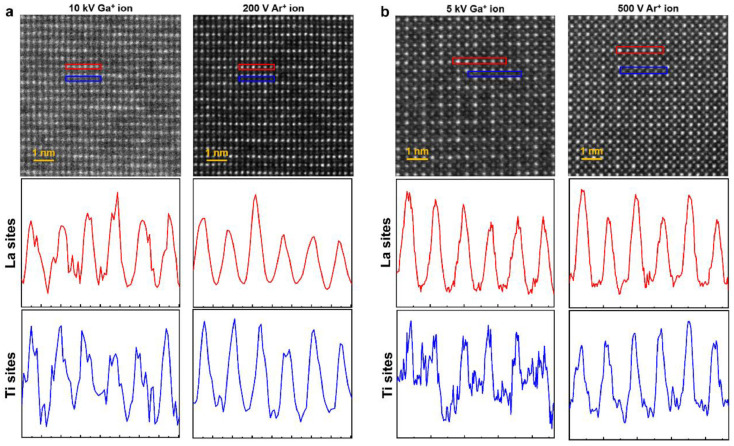
Effect of low-energy Ar^+^ milling on HAADF-STEM imaging of LLTO solid-electrolyte lamellae. HAADF-STEM images and corresponding intensity profiles of LLTO lamellae before and after low-energy Ar^+^ ion milling. (**a**) Specimen after 10 kV Ga^+^ ion milling and after subsequent 200 V Ar^+^ milling, viewed along the [201] zone axis. (**b**) Specimen after 5 kV Ga^+^ ion milling and after subsequent 500 V Ar^+^ milling, viewed along the [100] zone axis. Colored boxes mark the regions used for the intensity profiles. Adapted with permission from Ref. [32].

Solid-state electrolytes pose a different problem. Here, the key evidence often concerns light-element positions, local disorder, grain-boundary chemistry, alkali migration, or subtle interfacial decomposition. In the broader context of cryogenic and beam-sensitive energy-materials microscopy [30,31], these signals are easily affected by lamella surface quality. High-energy Ga^+^ milling can produce amorphous surface layers, implanted species, and artificial disorder. For lithium-site interpretation by ABF-STEM, iDPC, or related atomic-resolution methods, the same damaged surface layer can become decisive. In LLTO solid-electrolyte lamellae [32], post-FIB low-energy Ar^+^ milling reduces the damaged surface layer and improves STEM contrast, making weak atomic-column contrast more reliable for interpretation (Figure 4). For a Li-site or weak-column-contrast assignment, the minimum validation should include the amorphous-layer thickness before and after polishing, the final lamella thickness, the Ar^+^ milling conditions, and the electron dose used for imaging. Improved contrast after polishing demonstrates a reduction in one source of surface disorder; it does not by itself establish that the remaining contrast is free of preparation or dose effects. This improvement matters because weak atomic-column contrast and light-element-related information are sensitive to surface amorphization, thickness, contamination, and residual strain.

Mitigation should therefore be interpreted as a change in artifact profile rather than as a universal correction. Low-energy ion polishing can reduce one artifact channel, but ion energy, milling angle, exposure time, redeposition, preferential sputtering, and contamination all influence the final surface state [32]. For beam-sensitive solid electrolytes, the polishing step becomes part of the specimen-preparation history that must be considered during interpretation. A cleaner STEM image after polishing does not by itself prove that all preparation artifacts have been removed; it shows that one source of surface disorder has been reduced under a defined set of conditions. This distinction is important when the conclusion depends on lithium occupancy, oxygen sublattice distortion, grain-boundary segregation, or local structural disorder. Electron-beam effects add another layer of ambiguity in solid electrolytes. Both electron-beam imaging and Ga^+^ ion-beam exposure can induce Li or Na whisker growth in solid-state electrolytes, accompanied by chemical changes [9]. When such growth is linked to surface charging and can be suppressed by Au coating or cryogenic preparation, an alkali-rich protrusion observed after FIB-SEM handling cannot be assigned directly to electrochemical filament formation without considering SEM navigation, ion-beam exposure, and charge accumulation. Implanted species and beam-induced mixing represent a further source of false phase evidence. The IF-steel case [33] provides a clear mechanism for implanted-species chemistry: Ga introduced during FIB preparation can react with Fe to form crystalline FeGa_3_ with a defined orientation relationship to the substrate.

Modern energy systems contain many interfaces where this risk is realistic: current collectors, metallic interlayers, artificial SEI coatings, catalyst/electrolyte contacts, thin-film electrodes, and protective coatings on cathode particles. If the interface of interest is only a few to tens of nanometers thick, the spatial scale of preparation-induced mixing may be comparable to the feature being interpreted. A broadened interface, a Ga-containing region, a modified oxide shell, or a locally mixed layer may then be mistaken for an electrochemical reaction zone. Advanced STEM, EELS, or APT can measure such features with high precision, but high precision does not resolve their origin. The origin must be constrained by preparation route, ion species, dose, final polishing, transfer history, and comparison with alternative workflows.

The same issue appears in contemporary thin-film interface analysis. In a comparative study of heteroepitaxial Ag/Cu and Cu thin films, Ga-FIB preparation produced structural damage and elemental mixing and reduced the air stability of the prepared lamellae, whereas Xe-PFIB better preserved the interface structure and supported subsequent atomic-resolution imaging and in situ electrical-biasing experiments [34]. This comparison does not establish Xe-PFIB as universally damage-free. Instead, replacing Ga with Xe changes the dominant artifact profile: Ga-related implantation and chemistry may be reduced, while Xe implantation, sputtering behavior, curtaining, roughness, and final-polishing conditions remain relevant. Method selection should therefore be matched to the material and the information being interpreted rather than based on a general preference for one ion source.

The same reasoning applies across reactive energy materials. A claim about SEI chemistry requires control over liquid removal, cryogenic transfer, residual gas exposure, and analytical dose. A claim about alkali-metal filament growth requires control over charging, electron-beam exposure, and ion-beam-induced migration. A claim about lithium positions or local disorder in a solid electrolyte requires control over surface amorphization, lamella thickness, and low-Z signal stability. A claim about a new interfacial phase requires control over implanted species, local mixing, and beam-induced crystallization. The necessary control is determined by the claim being made. The minimum control should follow the chemical or structural assignment. SEI chemistry requires documented liquid removal, transfer conditions, residual exposure, and analytical dose. Li- or Na-rich filament formation requires controls for charging, electron-beam exposure, and ion-beam-induced migration. Lithium-site or local-disorder assignments require limits on surface amorphization, specimen thickness, contamination, and imaging dose. A newly identified interfacial phase requires tests for implanted-species chemistry, interface mixing, post-preparation oxidation, and beam-induced crystallization. These controls distinguish a feature belonging to the material from a chemically plausible feature generated during preparation or analysis.

## 5. Preparation Routes, Mitigation, and Claim-Specific Validation

Preparation-route selection should begin with the information being sought rather than with the instrument alone. A preparation method suitable for imaging cracks or heavy-element contrast in a dense oxide cathode may be unreliable for preserving lithium-metal chemistry or a reactive SEI. The same lamella may provide adequate high-angle annular dark-field scanning transmission electron microscopy (HAADF-STEM) contrast from heavy-element columns while remaining unsuitable for assigning lithium positions by annular bright-field or integrated differential phase-contrast STEM, because the latter measurements are more sensitive to surface amorphization, specimen thickness, contamination, and preparation-induced disorder. Similarly, a cross-section that preserves particle cracking may not preserve SEI chemistry. Preparation routes should therefore be judged by the information they preserve, the changes they may introduce, and the control available for the specific claim.

**Figure 5:**
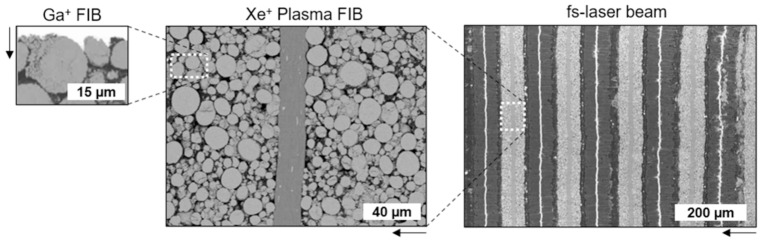
Cross-section dimensions accessible by Ga^+^ FIB, plasma FIB, and fs-laser preparation. Typical cross-section dimensions prepared by Ga^+^ FIB, plasma FIB, and fs-laser milling for battery-material analysis. Ga^+^ FIB provides site-specific small cross-sections, whereas plasma FIB and fs-laser-assisted milling extend accessible area for larger electrode or particle architectures. Arrows indicate the milling direction. Adapted with permission from Ref. [1].

Energy materials differ strongly in the structural or chemical information most readily altered during preparation and imaging. Stable oxide particles often tolerate conventional FIB preparation when the objective is crack morphology, coating continuity, or heavy-element mapping, although surface amorphization, oxygen loss, and dose-induced phase change may still limit atomic-scale interpretation. Lithium and sodium metals require tighter control of air exposure, residual gases, solvent removal, cryogenic transfer, and electron dose. Sulfide and halide solid electrolytes are sensitive to charging, radiolysis, volatilization, and chemical decomposition. Porous composite electrodes require reliable pore–binder contrast and segmentation, whereas metallic current collectors, interlayers, and thin-film contacts require control of implanted species, interfacial mixing, and post-preparation oxidation. A universal hierarchy of preparation methods is therefore not useful; the appropriate method is the one whose alteration profile is compatible with the information being claimed. The spatial scale of the evidence is also part of the preparation choice. Ga^+^ FIB, plasma FIB, and laser-assisted milling provide different balances between site specificity, preparation volume, throughput, and compatibility with subsequent TEM/STEM imaging, spectroscopy, tomography, or electrical testing [1]. A small Ga-FIB lamella may be ideal for atomic-resolution STEM of a selected interface, whereas mesoscale electrode cracking, pore-network heterogeneity, or representative-volume analysis may require larger cross-sections prepared by plasma FIB or laser-assisted routes. Figure 5 compares the typical cross-section dimensions obtained by Ga^+^ FIB, plasma FIB, and fs-laser milling [1], highlighting the link between preparation route and accessible evidence volume. Method selection therefore defines the evidence boundary before imaging begins, whether the claim concerns an atomic interface, a particle-scale degradation feature, or a mesoscale electrode architecture.

Conventional Ga-FIB lift-out [3,4] remains indispensable for targeted cross-sectioning and TEM/STEM specimen preparation. It provides high site specificity and is compatible with subsequent EDS, EELS, diffraction, and tomography. For many stable oxides, dense particles, and general solid–solid interfaces, it remains the most practical route. Its limitations are equally well known: amorphization, Ga implantation, redeposition, curtaining, local heating, roughness, and phase mixing [5,6,7,8]. These risks do not preclude Ga-FIB use, but they define the conditions under which the resulting evidence can be interpreted. Low-kV final polishing, protective capping, reduced ion current, and minimized exposure reduce risk only when they address the artifact channel relevant to the claim.

Cryogenic FIB and cryogenic lift-out are most appropriate when the target information is chemically fragile [25,26,29]. Alkali-metal electrodes, SEI layers, solid–liquid interfaces, liquid-containing regions, and some sulfide or halide electrolytes may be altered substantially by washing, drying, air exposure, or room-temperature transfer. Cryogenic preparation suppresses selected diffusion, evaporation, and reaction pathways, but it does not erase prior handling or prevent all subsequent change. Freezing quality, ice contamination, residual chamber chemistry, transfer time, local warming, charging, and analytical dose remain part of the final specimen state. Cryo-FIB should therefore be validated by documented transfer and temperature conditions and by low-dose or pre-/post-acquisition checks appropriate to the claimed feature.

Alternative ion sources and post-polishing methods should be evaluated by the claim they are intended to protect. Xe plasma FIB [34] can reduce Ga-specific implantation and chemical effects in some metallic interfaces and increase accessible volume, but interface stability should still be checked against Ga-FIB or an independently prepared reference where the conclusion depends on a nanometer-scale reaction layer. Low-energy Ar^+^ polishing [32] can reduce the amorphous surface layer of FIB-prepared solid-electrolyte lamellae, but its value should be demonstrated by before–after damaged-layer measurements and reported milling conditions. Broad ion-beam or ion-slicer preparation provides larger cross-sectional context at lower site specificity, requiring assessment of surface roughness and feature-location uncertainty. Ultramicrotomy is useful for soft or polymer-rich electrodes, but compression, tearing, knife marks, and particle pull-out should be evaluated. High-throughput particle-oriented FIB workflows [35] improve statistical access only when particle orientation, support geometry, thickness consistency, and the number of repeated particles are reported. Table 1 translates these material- and claim-dependent considerations into a practical assessment framework. For each representative structural or chemical claim, it identifies the preparation- or imaging-induced change that may produce a similar observation, the minimum control needed to distinguish the two origins, and the experimental information required for evaluation.

Reporting should be tied to the evidence being claimed. When preparation and acquisition parameters determine whether a signal can be assigned to the native material, ion species, beam energy, current, dose, milling angle, cap material, final polishing, temperature, transfer route, SEM conditions, TEM/STEM dose, and EDS/EELS acquisition conditions must be reported as part of the preparation and acquisition conditions used to evaluate the result. The required level of detail should then follow the claim: SEI chemistry depends strongly on transfer and dose history, lithium-site interpretation on damaged-layer control and lamella thickness, and pore tortuosity on segmentation criteria, voxel size, slice thickness, and representative volume.

Useful indicators include amorphous-layer thickness, implanted-ion signal, contamination and pre-/post-acquisition changes for lamella-based STEM; curtaining, slice-thickness variation, voxel anisotropy, pore-filling completeness, and segmentation-threshold sensitivity for tomography; and oxidation thickness, exposure-time dependence, exposure-time-dependent changes in elemental intensity or distribution, and phase change before and after mapping for reactive materials. Mitigation is most convincing when it is claim-specific. SEI chemistry places the burden on cryogenic transfer and low-dose mapping; lithium occupancy in solid electrolytes requires damaged-layer control and stable light-element imaging; electrode tortuosity depends on segmentation robustness and representative volume; and new interfacial phases require constraints on implanted-species chemistry, interface mixing, and beam-induced crystallization. Preparation should be selected after identifying the evidence most likely to be altered.

The broader implication is that mitigation belongs in the logic of interpretation, not in a methods appendix. A cryogenic stage, Xe ion source, low-kV polish, protective cap, or correlative dataset is valuable only when it addresses the artifact channel that could compromise the claim. For energy materials, preparation-route selection should therefore be judged by whether it preserves the specific structural, chemical, or morphological evidence on which the mechanism depends. In this form, mitigation becomes part of the interpretation rather than an isolated improvement in specimen preparation.

## 6. Outlook: From Artifact Avoidance to Evidence Attribution

FIB-enabled microscopy will remain central to the characterization of energy materials because many of the most important structures cannot be accessed from a free surface. Buried solid–solid contacts, reactive interphases, alkali-metal deposits, particle-scale cracks, thin protective coatings, current–collector interfaces, and porous electrode networks all require some form of controlled exposure before high-resolution imaging or spectroscopy becomes possible. The recent expansion of FIB-SEM into cryogenic preparation, plasma-FIB milling, correlative tomography, and multimodal battery characterization further strengthens its role. Its role is therefore expanding from specimen access to the conversion of hidden structural and chemical states into interpretable evidence. This position also makes reliability more demanding. A convincing FIB-enabled dataset cannot be judged only by image sharpness, spatial resolution, or the apparent consistency of an elemental map. The feature used to support the proposed transport, degradation, or interfacial mechanism must remain traceable to the material state under investigation. A lithium-rich region, oxide shell, reconstructed surface layer, interfacial compound, grain-boundary enrichment, or connected pore pathway may be real in the final specimen but still be shaped by handling, transfer, milling, imaging, or spectroscopy. For energy materials, where local chemistry and nanoscale morphology often carry most of the explanatory weight, this distinction determines whether microscopy supports a mechanism or simply records the history of making the specimen observable.

Future studies should therefore treat preparation and imaging history as part of the evidence, not as background information. The most useful controls will depend on the claim being made. Controls should address the structural or chemical feature most readily altered during preparation or imaging. SEI chemistry requires constraints on transfer, residual exposure, cryogenic handling, and analytical dose; solid-electrolyte light-element structure requires control of surface damage, lamella thickness, and beam sensitivity; electrode tortuosity requires constraints on sectioning artifacts, segmentation uncertainty, and representative volume; and interfacial phase assignment requires control of implanted species, ion-beam mixing, post-preparation oxidation, and dose-induced crystallization. The required control should be selective rather than exhaustive. Cryo-FIB, Xe-PFIB, low-energy ion polishing, and correlative tomography are most valuable when they address the artifact pathway most likely to alter the conclusion, whether that pathway involves reactive chemistry, Ga-related mixing, surface amorphization, or volume representativeness. FIB-enabled energy-materials microscopy should therefore move beyond the production of attractive images toward documented signal origin. Lower-damage preparation, improved detectors, cryogenic stages, faster milling, and automated reconstruction will improve the field, but technical refinement alone cannot remove interpretational ambiguity. A phase, interphase, elemental enrichment, light-element contrast, crack network, or pore descriptor becomes persuasive only when its path from native material to final dataset is documented well enough for the reader to judge what the microscopy result can and cannot prove.

## Figures and Tables

**Table 1:** Claim-specific controls for distinguishing intrinsic energy-material features from preparation- and imaging-induced changes.

Claimed Feature or Parameter	Representative Energy-Material Context	Workflow-Induced Confounder	Minimum Discriminating Control	Essential Reporting
Pore connectivity, tortuosity, or phase fraction	Porous composite electrodes analyzed by FIB-SEM tomography	Curtaining; redeposition; slice-thickness variation; pore/carbon-binder contrast overlap; threshold-dependent segmentation	Contrast enhancement or pore filling; threshold-sensitivity analysis; repeat or larger-volume reconstructions	Voxel size; measured slice thickness; drift correction; segmentation method and threshold range; analyzed volume
Coating continuity, crack width, or intraparticle damage	Coated cathode particles, secondary particles, and core–shell structures	Edge rounding; overmilling; shadowing; protective-cap masking; particle-orientation effects	Adjacent sections; multiple particles; alternative milling angle or cross-section geometry; low-kV final polish	Particle orientation; cap material/thickness; milling conditions; final specimen thickness; number of particles/regions
Li/Na-rich interphase, protrusion, or filament	Li/Na metals, SEI-bearing interfaces, and solid-state ion conductors	Air or moisture exposure; residual gases; charging; electron- or ion-beam-induced migration; local warming	Low-dose series; pre/post-imaging comparison; adjacent unexposed region; conductive coating where appropriate; cryogenic/room-temperature comparison	Transfer atmosphere and time; sample temperature; chamber history; SEM/TEM dose; ion species/current; electrochemical state
Light-element position or weak atomic-column contrast	LLTO and other oxide, sulfide, or halide solid electrolytes	FIB-induced amorphous layer; implanted ions; thickness variation; contamination; electron-dose damage	Before/after low-energy Ar^+^ polishing; damaged-layer measurement; thickness or dose series; repeat-image consistency	Final FIB energy/current; Ar^+^ energy, angle, and time; lamella thickness; zone axis; pixel size, dwell time, and dose
New interfacial phase or elemental enrichment	Metallic interlayers, current collectors, thin-film contacts, coatings, and catalyst/electrolyte interfaces	Ga implantation; interface mixing; local heating; beam-induced crystallization; post-preparation oxidation	Alternative ion source or final polish; unmilled/less-milled reference; depth-dependent composition; complementary analysis	Ion species and dose; cap layer; final polish; transfer atmosphere; time to analysis; EDS/EELS/APT acquisition conditions
SEI or solid–liquid-interface chemistry	Alkali-metal electrodes, liquid-containing interfaces, and reactive electrolytes	Electrolyte loss; washing/drying; freezing artifacts; ice contamination; residual oxidation; analytical dose	Controlled cryogenic transfer; low-dose sequence; no-wash or alternative preparation; room-temperature comparison when justified	Freezing method; transfer time and temperature; chamber conditions; washing/drying history; EDS/EELS dose and sequence
Residual strain, dislocations, bending, or crack opening in a lamella	Multilayers, brittle interfaces, coatings, and mechanically constrained electrode regions	Grid/post attachment; Pt/C weld shrinkage; asymmetric thinning; stress relaxation; membrane warping	Alternative attachment geometry; before/after-thinning imaging; thickness series; low-dose diffraction or strain mapping	Grid/post type; weld material and geometry; attachment sequence; thinning history; thickness gradient; specimen curvature
Surface carbon or altered surface chemistry after cleaning	Beam-sensitive interfaces, SEI, solid electrolytes, and spectroscopy specimens	Hydrocarbon deposition during SEM/TEM; plasma-induced oxidation, etching, or removal of volatile species	Pre/post-cleaning images or spectra; untreated reference; cleaning-condition series; low-dose verification	Cleaning gas; power; duration; pressure; time between cleaning and analysis; pre-existing beam exposure

## Data Availability

No new data were created or analyzed in this study.
